# Long-term outcome after combined or sequential liver and kidney transplantation in children with infantile and juvenile primary hyperoxaluria type 1

**DOI:** 10.3389/fped.2023.1157215

**Published:** 2023-03-17

**Authors:** Sebastian Loos, Markus J. Kemper, Kaja Schmaeschke, Uta Herden, Lutz Fischer, Bernd Hoppe, Tanja Kersnik Levart, Enke Grabhorn, Raphael Schild, Jun Oh, Florian Brinkert

**Affiliations:** ^1^ Department of Pediatric Nephrology, University Children's Hospital, University Medical Center Hamburg-Eppendorf, Hamburg, Germany; ^2^ Department of Pediatrics, Asklepios Klinik Nord Heidberg, Hamburg, Germany; ^3^ Department of Pediatric Gastroenterology and Hepatology, University Children's Hospital, University Medical Center Hamburg-Eppendorf, Hamburg, Germany; ^4^ Department of Hepatobiliary and Transplant Surgery, University Medical Center Hamburg-Eppendorf, Hamburg, Germany; ^5^ German Hyperoxaluria Center, c/o Kindernierenzentrum Bonn, Bonn, Germany; ^6^ Pediatric Nephrology, University Medical Center Ljubljana, Ljubljana, Slovenia; ^7^ Department of Pediatrics, University Children's Hospital, University Children's Research@Kinder-UKE, University Medical Center Hamburg-Eppendorf, Hamburg, Germany

**Keywords:** hyperoxaluria, infantile, juvenile, transplantation, outcome

## Abstract

**Introduction:**

Combined or sequential liver and kidney transplantation (CLKT/SLKT) restores kidney function and corrects the underlying metabolic defect in children with end-stage kidney disease in primary hyperoxaluria type 1 (PH1). However, data on long-term outcome, especially in children with infantile PH1, are rare.

**Methods:**

All pediatric PH1-patients who underwent CLKT/SLKT at our center were analyzed retrospectively.

**Results:**

Eighteen patients (infantile PH1 *n* = 10, juvenile PH1 *n* = 8) underwent transplantation (CLKT *n* = 17, SLKT *n* = 1) at a median age of 5.4 years (1.5–11.8). Patient survival was 94% after a median follow-up of 9.2 years (6.4–11.0). Liver and kidney survival-rates after 1, 10, and 15 years were 90%, 85%, 85%, and 90%, 75%, 75%, respectively. Age at transplantation was significantly lower in infantile than juvenile PH1 (1.6 years (1.4–2.4) vs. 12.8 years (8.4–14.1), *P *= 0.003). Median follow-up was 11.0 years (6.8–11.6) in patients with infantile PH1 vs. 6.9 years (5.7–9.9) in juvenile PH1 (*P* = 0.15). At latest follow-up kidney and/or liver graft loss and/or death showed a tendency to a higher rate in patients with infantile vs. juvenile PH1 (3/10 vs. 1/8, *P* = 0.59).

**Discussion:**

In conclusion, the overall patient survival and long-term transplant outcome of patients after CLKT/SLKT for PH1 is encouraging. However, results in infantile PH1 tended to be less optimal than in patients with juvenile PH1.

## 1. Introduction

One of the main indications for combined liver and kidney transplantation (CLKT) in children is primary hyperoxaluria type 1 (PH1) (1, 2). Although CLKT still is a challenging procedure, results in children are encouraging as shown by single-center studies and registry data from the United Network of Organ Sharing (UNOS) database, the Scientific Registry of Transplant Recipients (SRTR), the ESPN/ERA-EDTA-registry, and the OxalEurope registry (3–9). However, long-term outcome data are scarce and often analyses are not detailed enough to guide individual treatment in this rare procedure.

PH1 is a result of a defect of the peroxisomal liver enzyme alanine-glyoxylate-aminotransferase (AGT) (10). Oxalate deposition occurs in several tissues including the kidney. Clinical findings are kidney stones and/or nephrocalcinosis leading to chronic and end-stage kidney disease (CKD/ESKD) (10). Systemic oxalosis includes e.g., visual impairment, myocardial involvement, and oxalate osteopathy. PH1 is diagnosed *via* urine or plasma analysis for both oxalate and glycolate, and should be confirmed by genetic testing. Phenotypes can be divided in infantile, ESKD during the first year of life, and juvenile PH1. Especially children presenting with infantile PH1 have the highest burden of this devastating disease (11, 12).

Hydration, citrate supplementation, vitamin B6 (in those PH1-patients with susceptible *AGXT*-mutations), and, in case of ESKD, intensive kidney replacement therapy (KRT), and ultimately CLKT or sequential liver and kidney transplantation were the available treatment options until RNA interference (RNAi) therapeutics were developed (13).

Recently, a RNAi therapeutic was approved by the FDA and EMA [Lumasiran (OXLUMO), Alnylam, Cambridge, USA] for the treatment of PH1. Lumasiran and a second compound (Nedosiran, Dicerna, Lexington, USA) block endogenous oxalate production and lower plasma/urine oxalate (13–16). Lumasiran is approved for treatment in patients with estimated GFR (eGFR) ≥30 ml/min/1.73 m^2^ and was recently approved in ESKD (17, 18). However, long-term follow-up studies are not available yet. Isolated kidney transplantation under a RNAi therapeutic medication without liver transplantation (LT) might be possible in the future and first reports describing this strategy have been published (19). Results after isolated kidney transplantation in vitamin B6-sensitive patients might support this strategy (20).

We have previously reported favorable short-term results of children with PH1 and autosomal recessive polycystic kidney disease (ARPKD) undergoing CLKT in our center including children below <10 kg of body-weight (6, 21, 22). However, poorer long-term outcome due to a higher rate of surgical complications and the morbidity of the patients might be suspected in this age/weight-group especially in PH1.

We now aimed for an individual concise presentation of the overall long-term outcome of a large single- center cohort of children with PH1 and present the specific long-term outcome of patients with infantile PH1 compared to those with juvenile PH1.

## 2. Material and methods

### 2.1. Study design

A retrospective analysis of all pediatric patients with PH1 undergoing CLKT at the University Medical Center Hamburg between 1998 and 2020 was performed. Indication for CLKT was ESKD according to the local standard. One patient received a sequential liver and kidney transplantation (SLKT) but was included in the study. This patient was analyzed and presented together with patients with CLKT. Medical records were reviewed for clinical and routine laboratory data.

The following clinical definitions were applied: Infantile PH1 was defined by ESKD during the first year of life. Juvenile PH1 was defined by ESKD at an age >12 months. Liver graft failure was defined as timepoint of liver re-transplantation. Kidney graft failure was defined by re-initiation of KRT or kidney re-transplantation. eGFR was calculated by using the appropriate Schwartz formula (23, 24). Epstein-Barr virus (EBV)- positivity was defined by detection of EBV nuclear antigen (EBNA) immunoglobulin G (IgG) and/or EBV early antigen (EA) IgG.

The local ethical committee approved the study and informed consent was provided by patients/guardians (MC-068/11).

### 2.2. Statistical analysis

Data were analyzed using PRISM (Version 9, GraphPad, USA) or STATA (release 17, StataCorp, USA). Descriptive statistics are presented for continuous variables [median and interquartile range (IQR)] and for categorical variables (number and percentage). Continuous variables were compared using the Mann-Whitney *U* test. Fisher's exact test was used for categorical data. Survival curves were plotted using the Kaplan- Meier method. For multivariate analysis logistic regression was used. *P* values  < 0.05 were considered statistically significant.

## 3. Results

### 3.1. Cohort

Eighteen patients with PH1 underwent transplantation from 1998 to 2020 in our center, thereof 11 patients in the second part of the observation period from 2009 to 2020. The cohort comprised 17 patients with CLKT and one patient with SLKT (see below) which was included in the analysis for CLKT. In the following results, discussion and Supplementary Material all patients are summarized under CLKT.

PH1 was diagnosed by AGT-deficiency in the liver biopsy (*n* = 4) or identification of a specific mutation in the *AGXT*-gene (*n* = 14) (Supplementary Table S1). None of the patients with genetic testing had a homozygous classical vitamin B6-responsive genotype [c.508G > A, c.454T > A or c.731T > C (9)].

Main patient characteristics at transplantation (age, sex, body-weight, duration of KRT) are presented in Table 1. Ten patients (56%) reached ESKD during the first year of life and were thus classified as infantile PH1. All patients were on KRT before transplantation, either hemodialysis (HD) (*n* = 11), peritoneal dialysis (PD) (*n* = 1), or a combination of both (*n* = 6). Overall KRT was initiated at a median age of 0.6 years (0.2–14.1), at 0.3 years (0.26–0.4) in infantile PH1, and at 12.0 years (7.5–13.6) in juvenile PH1. Nine (50%) of all patients had a body-weight of less than 15 kg at the time of CLKT.

**Table 1:** Clinical data (median, IQR) of 18 patients with PH1.

		Whole cohort (*n* = 18)	Infantile PH1 (*n* = 10)	Juvenile PH1 (*n* = 8)	Infantile vs. juvenile PH1
At first CLKT	Age, years	5.4 (1.5–11.8)	1.6 (1.4–2.4)	12.8 (8.4–14.1)	*P *= 0.003
	Males, *n* (%)	10 (56)	6 (60)	4 (50)	*P *= 0.99
	Weight, kg	15.5 (10.0–28.8)	10.0 (9.2–13.0)	40.2 (22.8–52.0)	*P *< 0.001
	Duration of KRT, years	1.3 (0.8–1.7)	1.3 (1.0–1.6)	1.0 (0.3–1.8)	*P *= 0.56
	Donor/recipient-age-ratio, years	2.0 (1.5–6.7)	5.5 (1.9–21.1)	1.6 (0.8–2.0)	*P *= 0.036
	Donor/recipient-weight-ratio, kg	2.0 (1.1–5.4)	4.2 (1.2–6.6)	1.2 (0.8–2.8)	*P *= 0.96
	Liver graft/recipient-weight-ratio, kg	0.024 (0.017–0.033)	0.033 (0.029–0.041)	0.016 (0.013–0.019)	*P *< 0.001
Organ outcome after first CLKT	Follow-up period, years	9.2^a^ (6.4–11.0)	11.0^a^ (6.8–11.6)	6.9 (5.7–9.9)	*P *= 0.15
	Kidney and/or liver graft loss and/or death, *n* (%)	4/18 (22)	3/10 (30)	1/8 (13)	*P *= 0.59
	Kidney graft loss and/or eGFR <30 ml/min/1.73 m^2^, *n* (%)	6/17^a^ (35)	5/9^a^ (56)	1/8 (13)	*P *= 0.13
	Liver graft loss, *n* (%)	1/17^a^ (6)	1/9^a^ (11)	0/8 (0)	*P *> 0.99
	Height, *z*-score	−1.4^a^ (−2.4 to −0.7)	−1.7^a^ (−4.9 to −1.0)	−0.9 (−2.3 to −0.3)	*P *= 0.38
Organ function^e^ in patients w/o graft loss	Follow-up period, years	9.7^a^,^b^ (6.2–11.0)	11.0^a^,^b^ (6.0–12.0)	6.9 (5.7–9.9)	*P *= 0.15
	eGFR, ml/min/1.73 m^2^	53^a^,^b^,^c^,^d^ (38–70)	37^a^,^b^,^c^ (20–50)	68^d^ (57–75)	*P *= 0.038
	AST^f^, U/L	24^a^,^b^ (19–37)	23^a^,^b^ (15–50)	25 (18–38)	*P *= 0.86

AST, aspartate aminotransferase; CLKT, combined liver and kidney transplantation; eGFR, estimated glomerular filtration rate; KRT, kidney replacement therapy.

^a^ Fatal case excluded from analysis.

^b^ Data from patient 2 after third CLKT excluded.

^c^ Data from patient 3 after isolated kidney re-transplantation excluded.

^d^ Data from patient 1 after isolated kidney re-transplantation excluded.

^e^ At last follow-up.

^f^ Normal values by age: 1–12 year: <50 U/L, 13–19y: <30 U/L.

For the first CLKT, 16 patients received both organs from the same deceased donor. One patient received a living donation of both organs from a family member (patient 4, Supplementary Table S1). One patient with infantile PH1 underwent LT in another center 8 months before subsequent deceased donor kidney transplantation (patient 17, Supplementary Table S1). LT was performed using a whole organ (*n* = 6), a left lateral segment (*n* = 8), or a right extended lobe (*n* = 4).

Immunosuppression in most patients (11/17 (65%)) was based on tacrolimus, mycophenolate mofetil (MMF) ± prednisolone at latest follow-up. The individual immunosuppression is shown in Supplementary Table S2.

### 3.2. Patient survival

Overall, 17/18 patients survived (94%) after a median follow-up of 9.2 years (6.4–11.0) after first CLKT transplantation. One child with Down syndrome (patient 13, see below) died one week after CLKT due to infectious complications.

### 3.3. Organ function and survival

Of the 17 survivors 15 (88%) received KRT (continuous veno-venous hemo(dia)filtration [CVVH(D)F] or HD) for a median 10 days (5–21) after CLKT to lower the oxalate burden for the transplant kidney and/or to treat delayed graft function.

**Figure 1:**
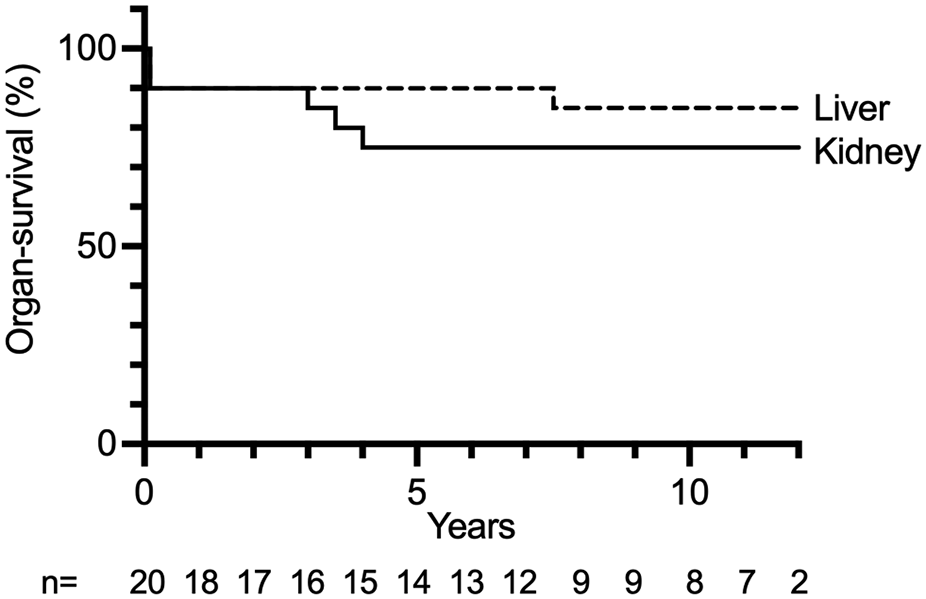
Liver and kidney graft survival after each CLKT performed. *n*: number of CLKTs under follow-up, censored at 12 years.

**Figure 2:**
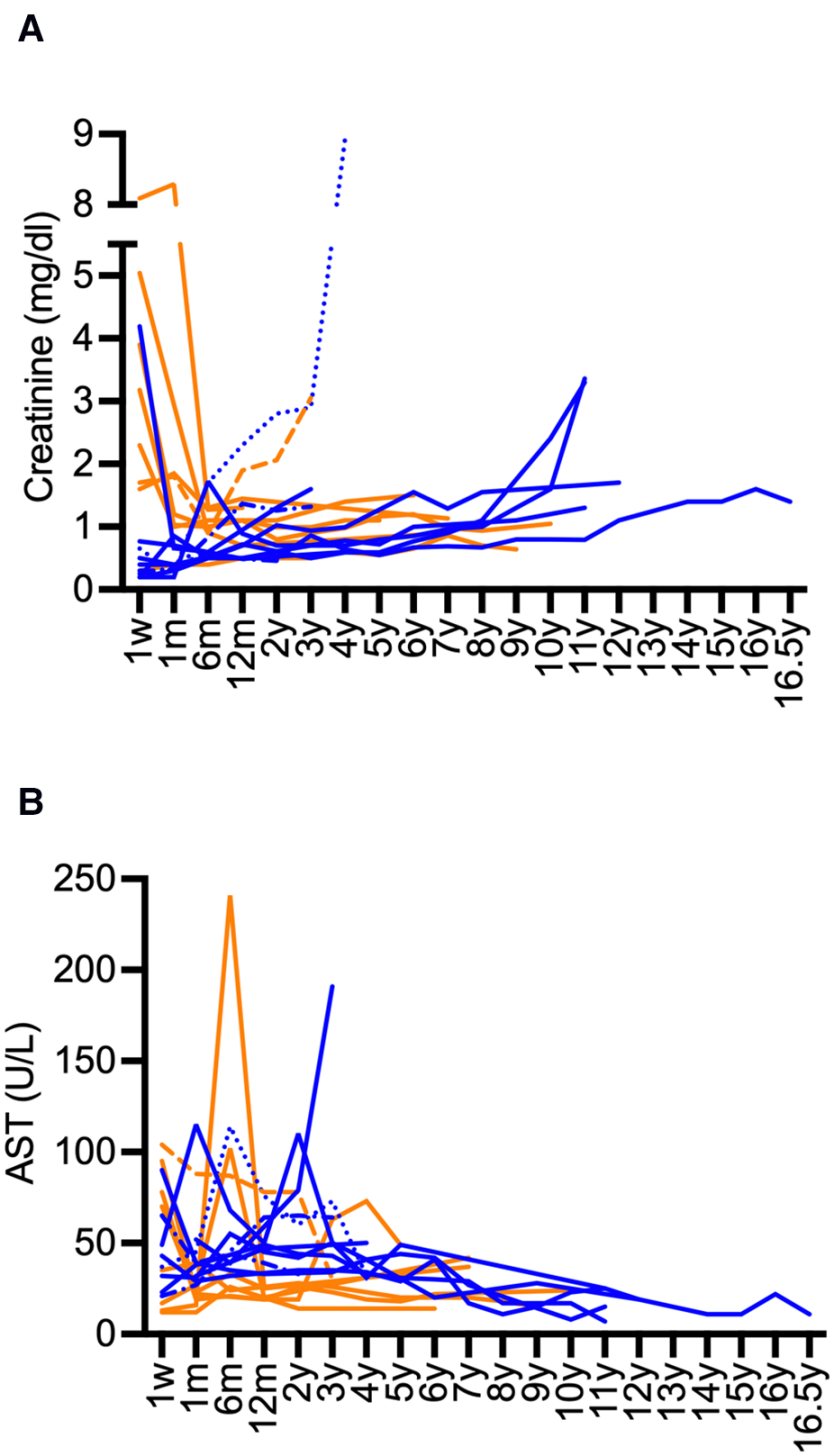
Long-term course serum-creatinine and AST after CLKT. (**A**): serum-creatinine (infantile PH1: blue, juvenile PH1: orange), (**B**): AST (infantile PH1: blue, juvenile PH1: orange); Patient 1 (-----) and patient 3 (······): creatinine values and AST after first CLKT are shown, patient 2 (-·-·-): data after second CLKT and third CLKT are shown, patient 13 (fatal case) is not shown.

Kidney graft survival after 1, 5, 10, and 15 years was 90%, 75%, 75%, and 75%, respectively (Figure 1). Liver survival after 1, 5, 10, and 15 years was 90%, 90%, 85%, and 85%, respectively (Figure 1). Overall long-term graft function after CLKT assessed by serum-creatinine and aspartate aminotransferase (AST) levels is shown in Figure 2.

In the multivariate analysis age at transplantation, weight at transplantation, donor/recipient-weight-ratio, donor/recipient-age-ratio, and liver graft/recipient-weight-ratio were not associated with liver or kidney graft loss (data not shown).

### 3.4. Complications after CLKT

Individual acute and chronic complications of all patients are presented in Supplementary Table S1.

Two children (patient 1 and 3) had to be re-started on KRT after 3.5 and 4.0 years, and they received another kidney transplant 7.0 and 6.0 years after CLKT, respectively. Patient 2 already needed a re-transplantation for both kidney and liver two weeks post CLKT because of primary kidney non-function and hepatic artery thrombosis. The same patient lost the second kidney after 3.0 years due to recurrent oxalate deposition in the transplant kidney after the citrate medication was removed from treatment at a tertiary center. This and chronic rejection of the liver resulted in the need of a third CLKT. In patient 13, CLKT was complicated by primary non-function of the liver despite normal perfusion of the graft. Thus, a secondary isolated LT was performed 4 days later. Further complications including acute respiratory distress syndrome (ARDS), fungal septicemia and abdominal ischemia occurred and the patient died 4 days later. Three of the four cases with re-transplantation and/or death occurred in the second part of the observation period from 2009 to 2020.

Four of the patients (22%) were EBV-serology-positive before CLKT. One patient, who was EBV-naïve at time of transplantation, developed EBV-positive post-transplant lymphoproliferative disease (PTLD) 11 years after transplantation and was successfully treated with an anti-CD 20 antibody (rituximab).

### 3.5. Outcome in patients with infantile vs. juvenile PH1

Data of the 10 patients with infantile PH1 compared to the 8 patients with juvenile PH1 are presented in Table 1. The baseline characteristics (duration of KRT, donor/recipient weight-ratio) at first CLKT were comparable, except of course for age, weight, and donor/recipient age-ratio.

Duration of follow-up was a median of 4.1 years longer in the infantile PH1. However, this was not statistically significant (Table 1). There was no statistically significant difference for death and/or kidney/liver graft loss between both groups (Table 1). However, 56% were affected in infantile PH1 vs. 13% in juvenile PH1. Accordingly, the proportion for the combined end-point kidney graft loss and/or eGFR <30 ml/min/1.73 m^2^ tended to be higher in patients with infantile vs. juvenile PH1 (Table 1). Results for the end-point liver graft loss were comparable (Table 1).

Patients with infantile PH1 showed a tendency towards a lower height *z*-score at latest follow-up (Table 1), which was not statistically significant.

After exclusion of patients with graft loss eGFR was lower in patients with infantile PH1 at latest follow-up (Table 1). AST at latest follow-up was comparable between both groups (Table 1).

### 3.6. Last follow-up

Median age at last follow-up was 15.3 years (12.5–19.4). Five patients were lost to further long-term follow-up because of treatment in another transplant center and/or transition to adult care. Latest serum-creatinine was 1.30 mg/dl (1.0–1.5) (age depended normal value 0.25–1.2 mg/dl), including patient 1 and 3 after isolated kidney re-transplantation and patient 2 after the third CLKT. At that timepoint none of the patients was on KRT. Median eGFR, AST, and height *z*-score (see above) are presented in Table 1.

### 3.7. Oxalate values after CLKT

**Figure 3:**
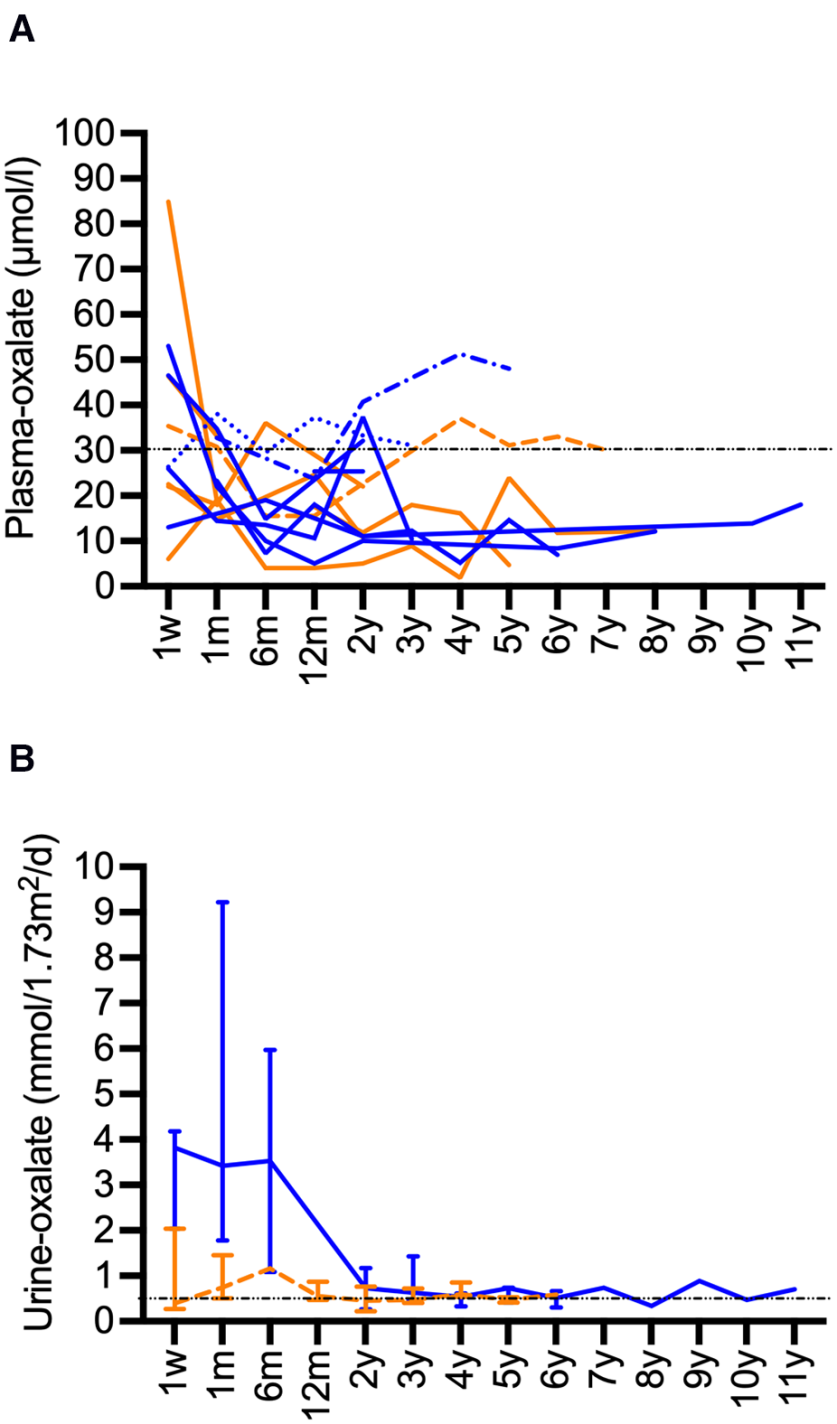
Long-term data for oxalate after CLKT. (**A**): Plasma-oxalate after CLKT (infantile PH1: blue, juvenile PH1: orange), patient 1 (------) and patient 3 (········): oxalate values after 1. CLKT transplantation are shown, patient 2 (-·-·-): data after second CLKT are shown, patient 13 (fatal case) is not shown, data for week 1 after transplantation might be compromised by KRT; (**B**): Urine-oxalate values (median and range, infantile PH1: blue, juvenile PH1: orange) in patients (infantile type *n* = 5, juvenile type *n* = 6) where two or more values were available.

Plasma-oxalate values decreased after CLKT. Available values are presented in Figure 3A. Most patients reached values below a target of 30 µmol/L over time. Higher values were only observed in those patients with subsequent need for KRT and kidney re-transplantation. Urine-oxalate remained above the target of <0.5 mmol/1.73 m^2^/d for a long period after CLKT (Figure 3B). Values <0.5 mmol/1.73 m^2^/d were reached after median 3 years (2–5) in 9 of 11 patients where replicate values were available. Especially in patients with infantile PH1 it persisted for years in some of the patients (Figure 3B).

## 4. Discussion

This study comprehensively presents long-term follow-up data of children that underwent CLKT due to PH1. In most cases patient and graft survival as well as growth up to 15 years after CLKT are encouraging. However, CKLT is a challenging procedure, which may be associated with severe complications including death. For the first time the long-term outcome in infantile PH1 is compared to juvenile PH1. Highly specialized treatment can lead to similar results in patients with infantile PH1 in comparison to patients with juvenile PH1.

### 4.1. Patient survival

The patient survival rate in our series is 94% after a median follow-up of 9.2 years and thus is not only superior to other series of patients with CLKT (4, 25). but also comparable to patients after isolated liver or isolated kidney transplantation for other indications (26, 27). As in our patient, mortality seems mainly to be associated to the post-operative period in CLKT (20).

The initial series of patients with CLKT by Jamieson et al. in 2005 showed a patient survival of 69% after 10 years. However, this report included children and adults (28). The analysis of the Scientific Registry of Transplant Recipients (SRTR) from Calinescu et al. from 2014 also showed a comparable 10-year patient survival of 65% (4). This study included 58 pediatric PH1 patients. Causes of death were mainly due to infections and cardiovascular complications. Our fatal case was due to primary non- functioning of the liver and a fungal infection leading to septicemia and multiorgan failure. Probably, the underlying condition of the patient (PH1 and Down syndrome) played a critical role in the clinical course. Data of 55 patients from the ESPN/ERA-EDTA-registry showed a survival rate of 76% after 5 years (8). Overall, various single-center reports demonstrated a better patient survival than registry data with a survival of up to 100%. For example, the Lyon group published their good experience with 100% survival in 14 PH1-patients after a median follow up of 3.8 years (0.5–13.3) (29). Büscher et al. presented data with a longer follow-up up to 10 years with a patient survival of 75% (5). The recently published data from the OxalEurope registry demonstrated a 10 year overall patient survival >80% for CLKT (20). In patients with infantile PH1 and CLKT the survival for up to 10 years follow-up was 80% in recent publication from the OxalEurope registry (9). However, lower patient survival rates are possible, especially in small centers with less routine (30). The most recent systematic review from Metry et al. could not present a meta-analysis regarding patient survival, due to under-reported survival probabilities with varying follow-up (25).

### 4.2. Organ function and survival

Kidney graft survival in PH1 patients is an important end-point, due to the risk of recurrence of oxalate deposition in the transplanted organ caused by the release of accumulated oxalate. It is well known that isolated kidney transplantation is overall inferior to combined transplantation with respect to kidney graft survival (25). The OxalEurope data showed that this paradigm might change for patients with B6-sensitve mutations. Even though in the OxalEurope-cohort overall kidney graft survival was superior in CLKT compared to isolated kidney transplantation, in those patients with B6-sensitive mutations patient survival was better and kidney graft survival comparable for isolated kidney transplantation vs. CLKT (20). However, the analysis was adjusted for duration of dialysis, age at ESKD, and age at transplantation.

The results of our study show a good long-term kidney survival with 75% after 10 and 15 years, respectively. This is remarkable, especially considering that more than 50% of the cohort are infantile PH1-patients with a high systemic oxalate burden. The registry data presented from Calinescu et al. showed a kidney survival of 50% after 10 years (4). Data from the OxalEurope registry show an overall 10-year overall kidney transplant survival of >80% and a 80% kidney survival in patients with infantile PH1 (9, 20). Some single-center studies observe a comparable outcome: The group from Lyon published a kidney survival of 79% in 14 PH1-patients after a follow up of 5 years (29). Büscher et al. showed a kidney survival of 4/5 (80%) in PH1-patients after CLKT after 11.5 years (5). These patients were transplanted between 8.0 and 17.8 years of age, which indicates that no infantile PH1-patients were in this group. In the kidney after liver program in infantile PH1-patients Büscher et al. reported a kidney survival of 75% (5). Metry et al. presented a similar range of kidney survival after 5 years between of 48%–89% in their review (25).

Liver graft survival was excellent in our cohort with 85% after 15 years. This shows, that the liver graft function can be stable over years once the challenging postoperative period, which is characterized by the risk of severe bleeding, hypotension, possible need for KRT, difficult volume management, severe infection, and cardiac morbidity, especially in patients with infantile PH1, is left behind. The results of our study are comparable to isolated LT with a survival of 79% (31). Data on isolated liver graft survival are infrequently reported in CLKT studies of PH1-patients. However, long-term survival rates of 60%–89% have been reported (4, 5, 9, 28).

Due to the retrospective study design and the small numbers of patients we cannot answer questions regarding the frequency of rejection in comparison of isolated liver or kidney transplantation vs. CLKT. Data in adults and children have shown that the liver might “immunologically protect” the kidney after sequential and CLKT (32, 33).

### 4.3. Complications after CLKT

Complications in this cohort are comparable to the results from isolated liver or kidney transplantations (27). Rejection episodes or infectious complications are common in all transplanted children.

Duclaux-Loras et al. reported that 7 out of 18 patients developed EBV-positivity with high viral loads and the need of lowering the immunosuppression or treatment with rituximab (29). One of these patients developed a Burkitt lymphoma. The immunosuppression seems to be comparable to our patients. However, in our cohort only one patient, who was EBV negative at time of transplantation, developed an EBV positive PTLD 11 years after CLKT and was successfully treated with rituximab. One explanation for this could be different trough levels of immunosuppressive medication and might explain the higher rate of EBV-infection/re-activation in the french analysis.

### 4.4. Outcome in patients with infantile vs. juvenile PH1 and last follow-up

Our own analysis regarding risk factors for complicated initial course after CLKT showed, that donor/recipient-age- and -weight-ratio play a role (21). Thus, carefully selected donors for pediatric patients are mandatory. As to be expected, in this study donor/recipient-age- and -weight-ratio were higher in patients with infantile compared to juvenile PH1. However, this difference was not statistically significant.

The rate regarding the outcome death or kidney/liver graft loss was higher in infantile PH1 and kidney outcome for the combined end-point kidney graft loss and/or eGFR <30 ml/min/1.73 m^2^ showed a tendency to an inferior outcome in infantile PH1. For both endpoints the difference was not statistically significant, which might be due to the low sample size. Accordingly, the eGFR in infantile PH1 patients was lower at last follow-up as discussed below. Liver survival and liver function were comparable between both groups at latest follow-up.

There are only two studies reporting outcome in small recipients (≤15 kg of body-weight, *n* = 8)/in infantile PH1 after CLKT (7, 9). The cohort by Perera et al. included 4 patients with PH1 and 4 patients with ARPKD. Base-line characteristics (age, weight, and donor-recipient-weight-ratio) of small recipients at first CLKT were comparable to our patients with infantile PH1. One patient out of 4 PH1-patients died due to septicemia and multi-organ failure. After a follow-up of 12 months median eGFR in small recipients was 53.8 ml/min/1.73 m^2^ compared to 64.9 ml/min/1.73 m^2^ in children >15 kg (*n* = 15) (*P* = 0.65) (7). Thus, kidney function was comparable in both groups at this timepoint. In our cohort kidney function was lower in infantile PH1 patients without re-transplantation. However, the overall follow-up was substantially longer in our cohort (median 9.7 years) and was median 4 years longer in infantile PH1 vs. juvenile PH1 However, this was not statistically significant. As mentioned above the data form the OxalEurope registry showed a good outcome for infantile PH1 after up to 10 years of follow-up: patient survival was 80%, kidney graft survival was 80%, and liver graft survival was above 60% (9).

### 4.5. Oxalate values after CLKT

Hyperoxaluria persists, especially in infantile PH1, over years after CLKT due to massive oxalate depositions especially the bone (9, 29). There is a high risk of recurrence in the transplant kidney. Our data show, that most patients with well-preserved kidney function reach plasma levels of oxalate below 30 µmol/L up to several months after CLKT. The patients with declining kidney function experienced rising plasma oxalate levels as even seen in non-PH1 ESKD patients. Urinary oxalate levels were elevated in some patients even after years. Therefore, alkaline-citrate medication should be continued.

In the future, the new treatment options with RNAi medication may lead way to a different approach of transplantation procedures in patients with PH1. LT might no longer be necessary with RNAi treatment, as it might be in selected patients with vitamin B6-responsive genotype (19, 20). Thus, further data are needed to advocate isolated kidney transplantation especially in children with PH1 under vitamin B6 and/or RNAi therapy. If such an approach might be possible in patients with infantile PH1, which experience rapid development of ESKD early in life, is questionable. In any case early diagnosis and initiation of the treatment with RNAi therapy will be crucial as this may prevent oxalate deposition in the body and might improve the overall outcome. This might highlight the importance of our data.

Our current study has some obvious limitations based on the retrospective study design and the single-center data acquisition. First of all, the patient numbers are small and this substantially affects the statistical analysis. Even though this is a single-center study, immediate post-operative management of the patients may have differed regarding fluid administration and/or KRT due to the long observation period of more than two decades. Furthermore, systemic oxalosis including cardiovascular disease and treatment including responsiveness to vitamin B6 before transplantation was not evaluated in a standardized manner. This might have an impact on short-term and long-term outcome due to differences in systemic oxalate burden between patients.

In conclusion, we present single-center results of severely affected PH1-patients who underwent combined liver and kidney transplantation. This study shows, that after the high-risk post-operative period, satisfactory results even in infantile PH1 can be achieved with the simultaneous transplantation strategy.

## Data Availability

The datasets presented in this article are not readily available because of legal restrictions due to data protection. Requests to access the datasets should be directed to the corresponding author.
