# Supplementary material for: Long-term outcome after combined or sequential liver and kidney transplantation in children with infantile and juvenile primary hyperoxaluria type 1
Source: Front Pediatr. 2023 Mar 17;11:1157215. doi: 10.3389/fped.2023.1157215 (PMC10064088; doi:10.3389/fped.2023.1157215)
Supplement: Supplementary file 1 [file Table1.docx]

**Supplementary Table 1.** Individual course of each patient

Chronic complications are defined as complications occurring and/or lasting longer than 3 months after CLKT

| Patient | Age at CLKT (years), sex, year of CLKT | Phenotype,  genotype/  AGT-activity | Prior | 1. Transplantation |  | 2. Transplantation |  | 3. Transplantation |  |
| --- | --- | --- | --- | --- | --- | --- | --- | --- | --- |
|  |  |  |  | Acute | Chronic | Acute | Chronic | Acute | Chronic |
| 1 | 8.0, f,  2007 | Juvenile PH1,  c.1079G>A | Pathologic fractures due severe bone disease | Vascular kidney rejection  Ductopenic liver rejection | ESKD and start of KRT after 3.5y for 3.5y  CMV-reactivation  Pulmonary tuberculosis | Isolated kidney transplantation after 7.0y  Plasmapheresis before transplantation due to HLA-antibodies  ABR | Recurrent UTIs |  |  |
| 2 | 2.3, f,  2011 | Infantile PH1,  c.1079G>A | Experimental Hepatocyte transplantation  *Oxalobacter formigenes*-therapy | Primary kidney non-function, hepatic artery thrombosis | n/a | High urgency re-CLKT after 2 weeks | Chronic kidney dysfunction  Start of KRT after 3y for 4.5y due to systemic oxalosis  Recurrent UTIs  Chronic liver rejection  Acute on chronic liver failure | CLKT after 7.5y  HLA-antibodies  Severe liver rejection with intensified immunosuppression and ATG-therapy | n/a |
| 3 | 1.4, m,  2010 | Infantile PH1  c.662_664del  CCT | n/a | Delayed kidney graft function  Acute liver rejection  Leakage of the bile duct anastomosis with the need of long-term stent insertion  CMV-reactivation | ESKD and start of KRT after 4y for 2 y | Isolated kidney transplantation after 6.0y  Cellular kidney rejection  CMV-reactivation | n/a |  | |
| 4 | 1.5, m,  2008 | Infantile PH1  compound heterozygous, c.331C>T, c.454T>A | n/a | CPR  Ureter-stenosis  Ileus | EBV-viremia  Chronic rejection and fibrosis liver |  | | | |
| 5 | 1.3, f,  2010 | Infantile PH1,  compound heterozygous, c.454T>A, c.846+1G>T | n/a | Acute rejection liver | BK-viraemia  Polyoma-nephropathy  EBV-associated PTLD 11y after transplantation |  | | | |
| 6 | 13, m,  2010 | Juvenile PH1,  c.519_520del  CCinsGA | n/a | n/a | Tremor |  | | | |
| 7 | 8.9, f,  1998 | Juvenile PH1,  Reduced activity | n/a | UTI  CMV-reactivation  Ascites due to small for size liver | n/a |  | | | |
| 8 | 1.6, m,  2007 | Infantile PH1,  compound heterozygous, c.508G>A, c.958delCA | n/a | CPR | UTI  Recurrent bacterial cholangitis |  | | | |
| 9 | 8.2, f,  2005 | Juvenile PH1,  Reduced activity | n/a | n/a | CMV-infection  Recurrent UTI  BK-viremia |  | | | |
| 10 | 12.6, m,  2012 | Juvenile PH1,  compound heterozygous, c.584T>G, c.614C>T | n/a | Diffuse liver bleeding  Leakage of the bile duct | Acute kidney and liver rejection |  | | | |
| 11 | 1.4, f,  2005 | Infantile PH1,  Reduced activity | n/a | Acute kidney rejection | ABR kidney  CMV-reactivation  Recurrent UTIs due to vesicoureteral reflux |  | | | |
| 12 | 2.8, m,  2003 | Infantile PH1,  Reduced activity | Isolated kidney transplantation in another center after diagnosis of dysplastic kidneys, graft loss after 2.5w due to recurrent oxalosis, subsequently diagnosis of PH1 | n/a | Bile duct stenosis |  | | | |
| 13 | 1.7, f,  2013 | Infantile PH1,  c.33dupC | Down Syndrome  Recurrent pulmonary infections | Primary liver non-function despite normal perfusion  Acute liver failure  ARDS  High urgency liver re-transplantation with good primary function  Severe septicaemia with subsequent necrosis of the intestine  Abdominal *Aspergillus*-infection  Exitus letalis | n/a |  | | | |
| 14 | 14.2, m,  2014 | Juvenile PH1,  c.33_34insC | n/a | Delayed kidney graft function  Ureter-stenosis  VZV-reactivation | Humoral and cellular kidney rejection |  | | | |
| 15 | 13.9, f,  2014 | Juvenile PH1,  compound heterozygous, c.508G>A, c.942+1G>T | Still’s disease treated in another center Eosinophilic pneumonia Lymphadenopathy Immunosuppression with steroids, Canakinumab, and Tocilizumab | Delayed kidney graft function | Car accident with multiple fractures and gut perforation  Recurrent UTIs |  | | | |
| 16 | 9.4, m,  2016 | Infantile PH1,  compound heterozygous, c.33dupC, c.364C>T | Severe systemic oxalosis (retinal, myocardial oxalosis, hearing loss, severe osteopathy) due to insufficient KRT (PD) over 9y  Start of additional HD in our center 6m prior to CLKT | Acute kidney rejection | UTIs |  | | | |
| 17 | 1.0, m,  2014 | Infantile PH1,  compound heterozygous, c.33insC, c.364C>T | Living-related liver transplantation 8m prior to kidney transplantation | En-bloc kidney transplantation  Unilateral nephrectomy after 2d due to primary non-function | BK-viruria  Hepatitis E-infection  Vesico-ureteral reflux |  | | | |
| 18 | 15.6, m,  2020 | Juvenile PH1,  compound heterozygous, c.508G>A,  c.847-3C>G | n/a | Urinoma with ureter obstruction | Liver rejection  HSV-reactivation  CMV-reactivation  Diabetes |  | | | |

ABR: Antibody-mediated rejection, AGT: Alanine- glyoxylate aminotransferase, ARDS: Acute respiratory distress syndrome, ATG: Anti-thymocyte-globulin, CLKT: Combined liver and kidney transplantation, CMV: Cytomegalovirus, CPR: Cardiopulmonary resuscitation, EBV: Epstein-Barr virus, ESKD: End-stage kidney disease, f: female, HD: Hemodialysis, HLA: Human leukocyte antigen, HSV: Herpes simplex virus, KRT: Kidney replacement therapy, n/a: not applicable, m: male, PD: Peritoneal dialysis, PTLD: Post-transplant lymphoproliferative disorder, UTI: Urinary tract infection, VZV: Varicella-zoster virus
