# Supplementary material for: Long-term outcome after combined or sequential liver and kidney transplantation in children with infantile and juvenile primary hyperoxaluria type 1
Source: Front Pediatr. 2023 Mar 17;11:1157215. doi: 10.3389/fped.2023.1157215 (PMC10064088; doi:10.3389/fped.2023.1157215)
Supplement: Supplementary file 2 [file Table2.docx]

**Supplementary Table 2.** Individual immunosuppression of each patient

Tac: tacrolimus, CyA: cyclosporin A, MMF: mycophenolate mofetil, Evero: everolimus, Pred: prednisolone, ATG: anti-thymocyte-globulin, Aza: Azathioprine

| Patient | 1. Transplantation | 2. Transplantation | 3. Transplantation | Last follow-up |
| --- | --- | --- | --- | --- |
| 1 | Basiliximab,  CyA, MMF, Pred | Tac, MMF, Pred |  | Tac, MMF, Pred |
| 2 | Tac, MMF, Pred | Tac, MMF, Pred | ATG,  Tac, MMF, Pred | Tac, MMF, Pred |
| 3 | Tac, Evero, Pred | Tac, Evero, Pred |  | Tac, Evero, Pred |
| 4 | Tac, Evero, Pred |  |  | Tac, Evero, Pred |
| 5 | Tac, MMF, Pred |  |  | Tac, Evero |
| 6 | Tac, MMF, Pred, |  |  | Tac, MMF |
| 7 | Tac, MMF, Pred , |  |  | Tac, Pred |
| 8 | CyA, MMF, Pred |  |  | Tac, Aza, Pred |
| 9 | Tac, MMF, Pred |  |  | Tac, MMF |
| 10 | Basiliximab,  CyA, MMF, Pred |  |  | Tac, MMF, Pred |
| 11 | CyA , MMF, Pred |  |  | Tac, MMF, Pred |
| 12 | CyA, MMF, Pred |  |  | Tac, MMF |
| 13 | Basiliximab,  Tac, Pred |  |  |  |
| 14 | Tac, Evero, Pred |  |  | Tac, MMF, Pred |
| 15 | Tac, MMF, Pred |  |  | Tac, MMF, Pred |
| 16 | Tac, MMF, Pred |  |  | Tac, MMF, Pred |
| 17 | Tac, Aza, Pred |  |  | Tac, Evero, Pred |
| 18 | Tac, MMF, Pred |  |  | Tac, MMF, Pred |
